# Bilateral and Unilateral Total Extraperitoneal Inguinal Hernia Repair (TEP) have Equivalent Early Outcomes: Analysis of 9395 Cases

**DOI:** 10.1007/s00268-015-3055-z

**Published:** 2015-04-02

**Authors:** F. Köckerling, C. Schug-Pass, D. Adolf, T. Keller, A. Kuthe

**Affiliations:** Department of Surgery and Center for Minimally Invasive Surgery, Vivantes Hospital, Academic Teaching Hospital of Charité Medical School, Neue Bergstraße 6, 13585 Berlin, Germany; StatConsult GmbH, Halberstädter Straße 40 a, 39112 Magdeburg, Germany; Department of General and Visceral Surgery, German Red Cross Hospital, Lützerodestraße 1, 30161 Hannover, Germany

## Abstract

**Introduction:**

To date, no randomized controlled trials have been carried out to compare the perioperative outcome of unilateral and bilateral inguinal hernia repair using an endoscopic technique. In a Swiss registry study comparing unilateral with bilateral inguinal hernias, no further details were given regarding the nature of the intra- and postoperative complications. In addition, some authors have raised the issue of prophylactic repair of a clinically healthy other groin side.

**Patients and methods:**

In the Herniamed Registry, in total 9395 patients with a TEP were enrolled. These comprised 6700 patients with unilateral (71.31 %) and 2695 patients (28.69 %) with bilateral inguinal hernia repair. The outcome variables, analyzed in a multivariable model, were the intra- and postoperative as well as general complication rates, reoperation rate, duration of operation, and length of hospital stay.

**Results:**

While no significant difference was found in the overall number of intraoperative complications between the unilateral and bilateral group (*p* = 0.310), a significantly higher number of urinary bladder injuries in the bilateral TEP operation of 0.28 % compared with 0.04 % for unilateral TEP (*p* = 0,008) were noted. The greater probability of reoperation (0.82 % for unilateral vs. 1.78 % for bilateral TEP; *p* < 0,001) in the unadjusted analysis was confirmed in the multivariable model [OR 2.35 (1.504; 3.322); *p* = 0.001].

**Summary:**

A significantly higher intraoperative urinary bladder injury rate and reoperation rate because of postoperative surgical complications constitute a difference in the perioperative outcome between unilateral and bilateral TEP which that warrants attention. Based on these results, prophylactic operation of the healthy other groin should not be recommended.

**Electronic supplementary material:**

The online version of this article (doi:10.1007/s00268-015-3055-z) contains supplementary material, which is available to authorized users.

## Introduction

The proportion of bilateral inguinal hernias identified using diagnostic laparoscopy is 28.5 % [1]. For bilateral inguinal hernia, all guidelines of the international surgical societies recommend laparoscopic/endoscopic repair in TAPP or TEP technique [2–5]. But to date no randomized controlled trials have been carried out to compare the perioperative outcome of unilateral and bilateral inguinal hernia repair using an endoscopic technique.

A Swiss registry study compared 3457 unilateral with 3048 bilateral inguinal hernia repairs using total extraperitoneal patch plasty (TEP) technique [6]. The authors identified an intraoperative complication rate of 1.9 % for unilateral and 3.1 % for bilateral TEP (*p* = 0.002). Likewise, the postoperative complications for unilateral TEP at 2.3 % and for bilateral TEP at 3.2 % were significantly different (*p* = 0.026). However, no further details were given regarding the nature of the intra- and postoperative complications. The authors concluded that the absolute difference between intra- and postoperative complications of unilateral versus bilateral inguinal hernia repair in TEP technique was small and of minor clinical relevance. In addition, some authors have raised the issue of prophylactic repair of a clinically healthy other side to avoid the second inguinal hernia repair in the future [7–9].

The following analysis of data from the Herniamed Registry [10] will now attempt to compare the perioperative outcome of unilateral with bilateral TEP, while also describing in greater detail the nature and severity of the intra- and postoperative complications and analyzing the influence factors. Only in this way can one really know whether extension of the indication to prophylactic operation of the healthy groin is justified.

## Patients and methods

Herniamed is a multicenter, internet-based hernia registry in which 358 participating clinics and surgeons in private practice from Germany, Austria, and Switzerland (status: April 2013) have prospectively registered their patients who had undergone hernia operation [10]. This present analysis now compares the prospective data of all patients who had undergone either unilateral or bilateral repair of an inguinal hernia in total extraperitoneal patch plasty (TEP) between 1 September 2009 and 15 April 2013. The inclusion criteria were a minimum age of 16 years, primary inguinal hernia, and uni- or bilateral TEP operation. In total, 9395 patients were enrolled. These comprised 6700 patients with unilateral (71.31 %) and 2695 patients (28.69 %) with bilateral inguinal hernia.

The TEP operations registered in the Herniamed Registry and now analyzed originated from 130 out of the 358 participating institutions. Thirty centers, each with more than 100 operations, accounted for 73.6 % of the surgical procedures. The remaining 100 centers thus provided data for 26.4 % of operations.

The demographic and surgery-related parameters included age (years), sex (m/f), ASA classification (I–IV) as well as the proportion of scrotal inguinal hernias, and the hernia defect size based on EHS classification (grade I–III). The outcome variables defined were the intra- and postoperative as well as general complication rates, reoperation rate, duration of operation, and length of hospital stay. Categorical data are presented as absolute and relative frequencies; continuous variables are displayed as mean, median, standard deviation, quantiles, and ranges. For the bilateral patient group, data on the variables given for both sides operated on were aggregated. For inguinal hernia defects of different sizes, the side with the larger defect is given. Classification as scrotal hernia was based on the presence of at least one scrotal hernia for bilateral inguinal hernia. Intra- and postoperative complications were recorded if a complication presented on at least one side. The same method was used to present details of any reoperation.

All analyses were performed with the software SAS 9.2 (SAS Institute Inc. Cary, NY, USA), and deliberately reviewed to the full level of significance. Each *p* value ≤ 0.05 thus represents a statistically significant result. To discern differences between the groups in unadjusted analysis, Fisher’s exact test was used for categorical outcome variables, and the *t* test for continuous outcome variables. For data that did not follow the normal distribution, as in the case of duration of operation and length of stay, the distribution was first transformed with the natural logarithm. To rule out any confounding of data caused by different patient characteristics, multivariable analyses were performed, while simultaneously reviewing other influence parameters in addition to laterality. To access influence factors in multivariable analysis, the general linear model was used for continuous, and the binary logistic regression model for dichotomous, numerical variables. Estimates for odds ratio (OR) or least square (LS) means, respectively, and the corresponding 95 % confidence interval were given. For age (years), the 10-year OR estimate was given. Patients (and not hernia) were the level of analysis.

## Results

### Unadjusted analysis

The mean patient age of 55.0 ± 16.0 years for unilateral inguinal hernia in TEP technique was significantly lower than for bilateral TEP at 56.1 ± 14.4 years (*p* = 0.0024). Likewise, the proportion of men at 87.49 % for unilateral inguinal hernia was lower than for bilateral inguinal hernia at 94.58 % (*p* < 0.001). ASA classification also revealed that for bilateral inguinal hernia a significantly greater proportion of patients belonged to the higher ASA classifications II–IV (*p* = 0.002). For the bilateral inguinal hernias, defect sizes of more than 3 cm, i.e., grade III as per EHS classification, were identified significantly more often, with fewer belonging to grade I (*p* < 0.001). The proportion of scrotal hernias in the bilateral group was significantly lower (*p* = 0.039). The results of the demographic and surgery-related parameters are given in Table 1.

**Table 1:** Demographic and surgery-related parameters

Demographic parameters	Unilateral	Bilateral	*p* value
Age			
Years ± StdDev	55.0 ± 16.0	56.1 ± 14.4	0.0024
Sex			
Male	5862 (87.49 %)	2549 (94.58 %)	<0.001
Female	838 (12.51 %)	146 (5.42 %)	
ASA score			
I	2206 (32.93 %)	799 (29.65 %)	0.002
II	3624 (54.09 %)	1573 (58.37 %)	
III	851 (12.70 %)	318 (11.80 %)	
IV	19 (0.28 %)	5 (0.19 %)	
Surgery-related parameters			
Hernia type			
Scrotal	132 (1.97 %)	36 (1.34 %)	0.039
EHS classification defect size			
Grade I (<1.5 cm)	1336 (19.94 %)	319 (11.84 %)	<0.001
Grade II (1.5–3 cm)	4094 (61.10 %)	1640 (60.85 %)	
Grade III (>3 cm)	1270 (18.96 %)	736 (27.31 %)	

*StdDev* standard deviation

The mean duration of operation for unilateral inguinal hernias in TEP technique was 44.7 min (range 20–275 min), and for bilateral inguinal hernias it was 60.3 min (range 20–270 min) (*p* < 0.0001). The mean length of stay for unilateral inguinal hernias was 1.6 days, and for bilateral inguinal hernias it was 1.8 days (*p* < 0.0001, range in both cases 1–63 days). Table 2 gives detailed results for duration of operation and length of stay.

**Table 2:** Duration of operation and length of stay

	Mean	Mean − StdDev	Mean + StdDev	*p* value
Duration of operation (min)				
Unilateral	44.7	30.0	66.7	*p* < 0.0001
Bilateral	60.3	40.9	89.1	
Length of stay (days)				
Unilateral	1.6	<1.0	2.6	*p* < 0.0001
Bilateral	1.8	1.1	3.0	

*StdDev* standard deviation

While no significant difference was found in the overall number of intraoperative complications between the unilateral and bilateral group (*p* = 0.310), a significant difference was noted for intraoperative organ injuries, which were higher for bilateral TEP operations (*p* = 0.018). That was essentially due to the significantly higher number of urinary bladder injuries in the bilateral TEP operation at 0.26 % compared with 0.04 % for unilateral TEP (*p* = 0.008) (Table 3).

**Table 3:** Intraoperative–postoperative complications and reoperations with unadjusted *p* values

Univariable analysis	Unilateral	Bilateral	*p* value
Intraoperative	80 (1.19 %)	39 (1.45 %)	0.310
Bleeding	53 (0.79 %)	16 (0.59 %)	0.351
Injuries (total)	42 (0.63 %)	30 (1.11 %)	0.018
Vascular	16 (0.24 %)	8 (0.30 %)	0.652
Bowel	4 (0.06 %)	1 (0.04 %)	1.000
Bladder	3 (0.04 %)	7 (0.26 %)	0.008
Nerve	1 (0.01 %)	0 (0.0 %)	1.000
Postoperative	114 (1.70 %)	49 (1.82 %)	0.727
Bleeding	77 (1.15 %)	25 (0.93 %)	0.380
Seroma	34 (0.51 %)	21 (0.78 %)	0.134
Intestinal lesion	0 (0.0 %)	1 (0.04 %)	0.287
Impaired wound healing	9 (0.13 %)	3 (0.11 %)	1.000
Infection	3 (0.04 %)	1 (0.04 %)	1.000
Intestinal obstruction	0 (0.0 %)	1 (0.04 %)	0.287
General	65 (0.97 %)	25 (0.93 %)	0.907
Exitus letalis	0 (0.0 %)	0 (0.0 %)	1.000
Reoperations	55 (0.82 %)	48 (1.78 %)	<0.001

As regards the postoperative surgical complications, no significant differences were found between unilateral and bilateral TEP in either the overall rate or in the individual parameters (Table 3).

Nonetheless, the reoperation rate because of perioperative complications at 1.78 % for bilateral TEP was significantly higher than for unilateral TEP at 0.82 % (*p* < 0.001) (Table 3).

No relevant differences were noted in the general postoperative complications (Table 3).

### Multivariable analysis

Model fit, reflecting the ability of the influence parameters to explain the outcome variable values, was not significant for intraoperative complications (*p* = 0.6369). Hence, individual variables were not found to have any significant impact on onset of intraoperative complications (Table 4).

**Table 4:** Multivariable analyses of intraoperative complications

Parameters	*p*	Category	OR estimate	95 % confidence interval	
				Lower CL	Upper CL
Defect size	0.246	I (<1.5 cm) vs. III (>3 cm)	1.285	0.72	2.285
		II (1.5–3 cm) vs. III (>3 cm)	0.864	0.55	1.369
Uni- vs. bilateral	0.287	Bilateral vs. unilateral	1.237	0.84	1.830
ASA score	0.543	II vs. I	1.217	0.77	1.919
		III vs. I	1.628	0.85	3.126
		IV vs. I			
Age (10-year OR)	0.863		1.012	0.88	1.161
EHS scrotal	0.894	Yes vs. no	0.907	0.22	3.784
Sex	0.959	Male vs. female	0.985	0.54	1.781

The model fit was not significant either for the general postoperative complications (*p* = 0.0617); therefore, it was not possible to identify any influence exerted by the model parameters on the occurrence of general complications. The only influence identified related to age, with higher age values tending to be associated with a higher rate of general complications (Table 5).

**Table 5:** Multivariable analyses of postoperative general complications

Parameters	*p*	Category	OR estimate	95 % confidence interval	
				Lower CL	Upper CL
Age (10-year OR)	0.028		1.203	1.02	1.418
Defect size	0.221	I (<1.5 cm) vs. III (>3 cm)	1.865	0.90	3.858
		II (1.5–3 cm) vs. III (>3 cm)	1.528	0.86	2.724
ASA score	0.272	II vs. I	1.223	0.70	2.131
		III vs. I	1.800	0.87	3.741
		IV vs. I	4.522	0.55	37.236
Sex	0.631	Male vs. female	1.189	0.59	2.405
EHS scrotal	0.700	Yes vs. no	1.330	0.31	5.671
Uni- vs. bilateral	0.955	Bilateral vs. unilateral	0.987	0.62	1.577

As in the unadjusted analysis, no significant difference was found between uni- and bilateral operations as regards the postoperative surgical complications [OR 1.040 (0.739; 1.464), *p* = 0.8277]. A higher ASA classification [*p* = 0.0099, for example ASA III vs. I; OR 1.737 (1.030; 2.931)], scrotal hernia [OR 1.749 (1.327; 5.695), *p* = 0.0065], and a higher patient age [10-year OR 1.177 (1.039; 1.334), *p* = 0.0107] were conducive to onset of postoperative complications (Table 6).

**Table 6:** Multivariable analyses of postoperative surgical complications

Parameters	*p*	Category	OR estimate	95 % confidence interval	
				Lower CL	Upper CL
EHS scrotal	0.006	Yes vs. no	2.749	1.33	5.695
ASA score	0.010	II vs. I	0.923	0.61	1.391
		III vs. I	1.737	1.03	2.931
		IV vs. I	3.731	0.79	17.621
Age (10-year OR)	0.011		1.177	1.04	1.334
Defect size	0.092	I (<1.5 cm) vs. III (>3 cm)	0.613	0.32	1.159
		II (1.5–3 cm) vs. III (>3 cm)	1.142	0.78	1.672
Sex	0.195	Male vs. female	1.512	0.81	2.825
Uni- vs. bilateral	0.823	Bilateral vs. unilateral	1.040	0.74	1.464

The greater probability of reoperation for bilateral operations, as already demonstrated by unadjusted analysis, was confirmed in the multivariable model (*p* = 0.0001). The odds ratio of bilateral vs. unilateral TEP was 2.35 (1.504; 3.322). Besides, a higher ASA classification was likewise conducive to reoperation [*p* = 0.0003, for example ASA III vs. I; OR 2.121 (1.120; 4.017)], whereas none of the other potential influence variables had a significant impact on the need for reoperation (Table 7).

**Table 7:** Multivariable analyses of reoperations for complications

Parameters	*p*	Category	OR estimate	95 % confidence interval	
				Lower CL	Upper CL
Uni- vs. bilateral	<.001	Bilateral vs. unilateral	2.235	1.50	3.322
ASA score	<.001	II vs. I	0.811	0.49	1.343
		III vs. I	2.121	1.12	4.017
		IV vs. I	8.344	1.73	40.345
EHS scrotal	0.190	Yes vs. no	2.027	0.70	5.836
Age (10-year OR)	0.566		1.046	0.90	1.222
Defect size	0.608	I (<1.5 cm) vs. III (>3 cm)	0.846	0.41	1.755
		II (1.5–3 cm) vs. III (>3 cm)	1.138	0.70	1.846
Sex	0.896	Male vs. female	1.048	0.52	2.113

Onset of postoperative secondary bleeding was influenced by a higher ASA classification [*p* = 0.0003, for example ASA III versus I; OR 2.747 (1.410; 5.338)] as well as higher patient age [10-year OR 1.208 (1.028; 1.418), *p* = 0.0217] (Table 8).

**Table 8:** Multivariable analyses of postoperative bleeding

Parameters	*p*	Category	OR estimate	95 % confidence interval	
				Lower CL	Upper CL
ASA score	<.001	II vs. I	1.110	0.64	1.935
		III vs. I	2.743	1.41	5.338
		IV vs. I	8.340	1.68	41.303
Age (10-year OR)	0.022		1.208	1.03	1.418
Defect size	0.072	I (<1.5 cm) vs. III (>3 cm)	0.826	0.37	1.869
		II (1.5–3 cm) vs. III (>3 cm)	1.553	0.93	2.584
EHS scrotal	0.223	Yes vs. no	1.940	0.67	5.632
Sex	0.324	Male vs. female	1.449	0.69	3.026
Uni- vs. bilateral	0.343	Bilateral vs. unilateral	0.801	0.51	1.267

The proportion of postoperative seromas rose significantly in the presence of a scrotal inguinal hernia [OR 4.390 (1.644; 11.719), *p* = 0.0032] (Table 9).

**Table 9:** Multivariable analyses of postoperative seroma

Parameters	*p*	Category	OR estimate	95 % confidence interval	
				Lower CL	Upper CL
EHS scrotal	0.003	Yes vs. no	4.390	1.64	11.719
Sex	0.119	Male vs. female	4.858	0.67	35.491
Uni- vs. bilateral	0.228	Bilateral vs. unilateral	1.403	0.81	2.435
Defect size	0.263	I (<1.5 cm) vs. III (>3 cm)	0.398	0.13	1.221
		II (1.5–3 cm) vs. III (>3 cm)	0.763	0.42	1.399
Age (10-year OR)	0.386		1.097	0.89	1.352
ASA score	0.996	II vs. I	1.024	0.53	1.989
		III vs. I	0.923	0.34	2.478
		IV vs. I			

The highly significantly impact of uni- and bilateral TEP on the duration of operation (*p* < 0.0001), as already revealed by unadjusted evaluation, was confirmed in the multivariable model [46.68 min (44.39; 49.08) for unilateral and 62.29 min (59.10; 65.65) for bilateral operation]. Furthermore, a large defect size, scrotal hernia as well as operation for a male patient (in each case *p* < 0.0001) resulted in significant prolongation of the duration of operation. Conversely, as regards ASA classification (*p* = 0.0050), longer durations of operations were required for a lower classification (Table 10).

**Table 10:** Multivariable analyses of operation time

Parameters	*p*	Category	LS mean estimate	95 % confidence interval	
				Lower CL	Upper CL
Uni- vs. bilateral	<.001	Unilateral	46.68	44.39	49.08
		Bilateral	62.29	59.10	65.65
EHS scrotal	<.001	Yes	60.72	56.48	65.28
		No	47.88	45.90	49.94
Sex	<.001	Male	56.24	53.53	59.09
		Female	51.69	48.92	54.63
Defect size	<.001	I (<1.5 cm)	53.02	50.23	55.96
		II (1.5–3 cm)	52.88	50.22	55.69
		III (>3 cm)	55.91	53.10	58.87
ASA score	0.005	I	57.56	55.52	59.67
		II	56.03	54.16	57.97
		III	55.53	53.36	57.79
		IV	47.20	40.19	55.44
Age (ML estimate)	0.449		0.002	−0.004	0.008

Die postoperative length of stay rose significantly for bilateral operation [*p* < 0.0001, 2.14 days (2.01; 2.28) for unilateral and 2.48 days (2.33; 2.65) for bilateral repair]. A larger defect size (*p* = 0.0001), higher ASA classification as well as scrotal inguinal hernia (in each case *p* < 0.0001) likewise prolonged length of stay. Furthermore, postoperative hospital stay was significantly longer for women than for men (*p* < 0.0001) (Table 11).

**Table 11:** Multivariable analyses of hospital stay

Parameters	*p*	Category	LS mean estimate	95 % confidence interval	
				Lower CL	Upper CL
Uni- vs. bilateral	<.001	Unilateral	2.14	2.01	2.28
		Bilateral	2.48	2.33	2.65
ASA score	<.001	I	1.90	1.81	1.98
		II	1.98	1.90	2.07
		III	2.27	2.16	2.39
		IV	3.30	2.71	4.03
EHS scrotal	<.001	Yes	2.62	2.39	2.87
		No	2.03	1.93	2.14
Sex	<.001	Male	2.19	2.06	2.33
		Female	2.42	2.26	2.59
Defect size	<.001	I (<1.5 cm)	2.35	2.20	2.51
		II (1.5–3 cm)	2.34	2.20	2.50
		III (>3 cm)	2.22	2.08	2.37
Age (ML estimate)	0.299		0.004	−0.004	0.012

Multivariable analysis was able to verify the differences identified in the unadjusted tests between uni- and bilateral TEP. According to that, on taking into account the patient and operation characteristics, both the duration of operation and postoperative length of stay were significantly longer for bilateral compared with unilateral TEP operation. Likewise, the reoperation rate because of perioperative complications was higher for bilateral than for unilateral TEP operation. The complication rates were not significantly influenced by bilateral TEP operation.

## Discussion

The present analysis compared early postoperative outcome of 6700 unilateral with 2695 bilateral TEP operations from the Herniamed Registry. While no difference was found in the overall number of intraoperative complications, detailed analysis revealed a significantly higher rate of urinary bladder injuries for bilateral TEP operations. Also, no significant difference was detected in the overall rate of postoperative surgical complications. But apparently the severity of postoperative surgical complications differed since the reoperation rate of 1.78 % for bilateral TEP was significantly higher than for unilateral TEP at 0.82 % (*p* < 0.001). The odds ratio of bilateral vs. unilateral TEP was 2.24 [1.50; 3.32]. Only a higher ASA classification was likewise conducive to reoperation (*p* = 0.0003), whereas none of the other potential influence variables exerted a significant impact on the need for reoperation.

However, when evaluating these results, it must be borne in mind that during the around three-and-a-half-year survey period 100 hospitals contributed data on fewer than 100 surgical procedures. As such, the majority of participating institutions must be classified as low-volume hospitals with regard to the TEP operation. That reflects the reality in the participating countries.

As regards the significantly higher reoperation rate for bilateral TEP, we also noted the same for bilateral transabdominal preperitoneal patch plasty (TAPP) in a separate analysis of the Herniamed data. Comparison of 10,887 unilateral with 4289 bilateral TAPP operations also revealed a significantly higher reoperation rate (0.9 vs. 1.9 %; *p* = < 0.001). But for TAPP, too, 138 of the 181 hospitals that contributed data on TAPP operations to the Herniamed Registry had performed fewer than 100 procedures during the three-and-a-half-year survey period. Again, it was mainly low-volume hospitals that supplied the data for the TAPP operations. That must be taken into consideration when evaluating the results.

As regards the general postoperative complications, no significant differences were discerned between unilateral and bilateral TEP. The significantly longer durations of operation and lengths of hospital stay following bilateral TEP compared with unilateral TEP turned out to be as expected.

As such, the present comparative registry study based on a large patient collective reveals that there are relevant differences in patient perioperative outcome between uni- and bilateral TEP. A significantly higher intraoperative urinary bladder injury rate and a significantly higher reoperation rate because of postoperative surgical complications constitute a difference in the perioperative outcome between unilateral and bilateral TEP which that warrants attention. No doubt that higher risk to patients can be countenanced if the patient presents with a bilateral inguinal hernia.

A higher complication rate resulting from repair of bilateral inguinal hernia in a single operation could be accepted provided that this is on a reasonable scale. Repair of bilateral inguinal hernia in two separate operations is also likely to result in a higher complication rate.

Prophylactic operation of the other groin in the case of unilateral inguinal hernia, as recommended by some surgeons [7–9] should, however, be viewed in a very critical light on the basis of the data presented here. That can be carried out only by highly experienced endoscopic surgeons, with an extremely low complication rate, and should definitely not be recommended in general. The potential risks to the individual patient of an increased urinary bladder injury rate and higher reoperation rates are too serious to justify that approach. Above all, these significantly higher perioperative complications are likely to give rise to increased rates of chronic pain in the later course.

The main focus of the present study was to analyze in detail the perioperative outcomes in order to in turn compare these findings with the data in the literature. On completion of follow-up, we intend analyzing the recurrence rate and the chronic pain rate at a later date.

Therefore, as before, in compliance with the guidelines [2–5] bilateral inguinal hernia should preferably be repaired using an endoscopic/laparoscopic technique. Any intraoperatively diagnosed occult inguinal hernias should also be repaired at the same time. It is therefore always advisable to inform patients about that possibility. But when providing information on bilateral operation, reference must be made in future to the increased risk of bladder injury and of the need for reoperation for TEP. That applies even more so if an experienced endoscopic surgeon continues to recommend to patients, with unilateral inguinal hernia, prophylactic operation of the other side. Based on the results presented here, prophylactic operation of the healthy other groin should not be recommended in general for patients with unilateral inguinal hernia.

## Electronic supplementary material

Supplementary material 1 (DOC 40 kb)
